# Thymoquinone Ameliorates Lung Inflammation and Pathological Changes Observed in Lipopolysaccharide-Induced Lung Injury

**DOI:** 10.1155/2021/6681729

**Published:** 2021-03-30

**Authors:** Mostafa Boskabady, Mohammad Reza Khazdair, Rahimeh Bargi, Saeideh Saadat, Arghavan Memarzia, Nema Mohammadian Roshan, Mahmoud Hosseini, Vahid Reza Askari, Mohammad Hossein Boskabady

**Affiliations:** ^1^Applied Biomedical Research Center, Mashhad University of Medical Sciences, Mashhad, Iran; ^2^Department of Physiology, Faculty of Medicine, Mashhad University of Medical Sciences, Mashhad, Iran; ^3^Cardiovascular Diseases Research Center, Birjand University of Medical Sciences, Birjand, Iran; ^4^Department of Physiology, School of Medicine, Zahedan University of Medical Sciences, Zahedan, Iran; ^5^Department of Pathology, Faculty of Medicine, Mashhad University of Medical Sciences, Mashhad, Iran; ^6^Division of Neurocognitive Sciences, Psychiatry and Behavioral Sciences Research Center, Mashhad University of Medical Sciences, Mashhad, Iran; ^7^Department of Pharmaceutical Sciences in Persian Medicine, School of Persian and Complementary Medicine, Mashhad University of Medical Sciences, Mashhad, Iran

## Abstract

Anti-inflammatory, antioxidant, and immunomodulatory effects of thymoquinone (TQ) have been shown. The effects of TQ on lipopolysaccharide- (LPS-) induced inflammation and pathological changes in rats' lung were investigated in this study. Four groups of rats included (1) control (saline treated); (2) LPS (treated with 1 mg/kg/day i.p. for two weeks); and (3 and 4) 5 or 10 mg/kg TQ i.p. 30 min prior to LPS administration. Total and differential WBC counts in the blood and bronchoalveolar fluid (BALF), TGF-*β*1, INF-*γ*, PGE2, and IL-4 levels in the BALF and pathological changes of the lung were evaluated. Total WBC count and eosinophil, neutrophil, and monocyte percentage were increased, but the lymphocyte percentage was reduced in the blood and BALF. The BALF levels of PGE2, TGF-*β*1, and INF-*γ* were also increased, but IL-4 level was reduced due to LPS administration. LPS also induced pathological insults in the lung of rats (*P* < 0.05 to *P* < 0.001 for all changes in LPS-exposed animals). Treatment with TQ showed a significant improvement in all changes induced by LPS (*P* < 0.05 to *P* < 0.05). TQ showed a protective effect on LPS-induced lung inflammation and pathological changes in rats which suggested a therapeutic potential for TQ on lung injury.

## 1. Introduction

Inflammatory response, under normal conditions, is a self-limiting process, but in several chronic diseases including lung injury, it could result in prolonged inflammation and tissue injury [1, 2]. It is well documented that overproduction of ROS in oxidative stress resulted in lung damage by various processes, including inflammation of the airways, which contribute to the pathogenesis and/or exacerbation of airways diseases [3] and the interrelationship between enhanced ROS generation and tissue inflammation [4].

Lung injury is commonly associated with endotoxemia, the presence of lipopolysaccharide (LPS) in the bloodstream; however, the mechanism that endotoxin induces the inflammatory response in acute lung injury is not well defined. Several studies reported inflammation in different organs induced by systemic administration of LPS, which is due to the production of reactive oxygen species (ROS) and proinflammatory cytokines [5, 6]. Our previous studies showed that intraperitoneal (i.p.) administration of LPS in the rats increased total and differential WBC count, induced oxidative damage by increasing the MDA level and decreasing antioxidant markers, and led to a Th1/Th2 imbalance in the blood and the BALF [7–10]. Therefore, reducing oxidative injury and suppression of the inflammatory process could ameliorate LPS-induced tissue damage including lung injury.

In several animal models of lung injury, inflammation and oxidative stress are involved as the underlying pathophysiological mechanisms. Thus, anti-inflammatory or antioxidant agents have been widely used to alleviate lung injury. Thymoquinone (TQ), the main component of *Nigella sativa* (*N. sativa*) seeds, showed anti-inflammatory and antioxidant effects in various conditions including bronchial asthma.

Various studies indicated that administration of TQ ameliorated tracheal responsiveness to methacholine and ovalbumin, as well as total and differential WBC, especially eosinophilia [11], improved Th1/Th2 balance by enhancing the IL-4 but reducing IFN-*γ* levels, and improved pathological changes of the lung in the animal models of asthma [12–16]. Preventive and therapeutic effects of TQ in cyclophosphamide-induced lung injury in rats [17] and LPS-induced hepatotoxicity in mice [18] were also reported. In addition, TQ inhibited LPS-induced inflammatory mediators in BV2 microglial cells [19] and increased the expression of neuroprotective proteins and decreased the expression of proinflammatory cytokines and the gene expression of NF*κ*B pathway signaling targets in LPS/IFN*γ*-activated BV-2 microglia cells [20]. TQ also suppressed production of Th2-type cytokines by mast cells in response to LPS stimulation in *vitro* [21].

Therefore, in this study, the effect of TQ on LPS-induced lung injury was examined. For this purpose, lung pathological changes, levels of cytokines in bronchoalveolar lavage fluid (BALF), and total and differential white blood cells (WBCs) counts in the BALF and blood of rats were evaluated in control, LPS-administrated, and LPS groups treated with TQ.

## 2. Materials and Methods

### 2.1. Animals and Drugs

Wistar rats (male < weighing 240 ± 10 g) were purchased from the animal house, Mashhad University of Medical Sciences, Mashhad, Iran, and kept in the same place in groups of 3 in individually ventilated cages with free access to food and water. Animals were maintained under temperature of 22 ± 2°C, relative humidity of 54 ± 2%, and 12 h light/dark cycles. The ethics committee of Mashhad University of Medical Sciences approved animal experimental protocols (Project ID 971117, 2 July 2017). Four groups of rats (*n* = 6 in each group) were studied as described in Table 1.

### 2.2. Blood and BALF Collection

The rats were sacrificed after deeply anesthetizing by 1.6 g/kg intraperitoneal (i.p.) administration of urethane at the end of the two weeks. The animals were sacrificed by a competent researcher with a minimum pain, suffering, and distress. The method was performed according the Annex IV of the guidelines from Directive EU/2010/63 of the European Parliament guideline. The blood samples were obtained by cardiac puncture. The blood samples were dispensed into the anticoagulant-containing tubes to be used for WBC count.

To prepare BALF samples, 1 ml phosphate buffered saline (PBS) was injected through a cannula inserted into the trachea of the right lung and was then aspirated. The procedure was repeated 5 times (total volume of 5 ml) [22, 23].

After sample collection, the heart of the rats was removed to euthanize the animals. The sample collection and euthanization of the animals were performed by a competent researcher with a minimum pain, suffering, and distress. The method was performed according the Annex IV of the guidelines from Directive EU/2010/63 of the European Parliament guideline [23, 24].

### 2.3. Blood and BALF Total and Differential WBC Counts

The cell pellets were suspended in normal saline after centrifuging BALF at 2500 rpm at 4°C for 10 min, and total and differential cell counts were measured.

Total WBC count was measured in 1 ml of blood or BALF stained with Turk's solution using a Neubauer counting chamber. Differential count of WBCs was determined in a smear of the blood or BALF stained with Wright–Giemsa, as described previously, under a light microscope [25].

### 2.4. Measurement of Cytokine Levels in the BALF

Interleukin-4 (IL-4), interferon-gamma (IFN-*γ*), transforming growth factor-beta-1 (TGF-*β*1), and prostaglandin-E2 (PGE2) levels in the BALF were measured by the specific ELISA kits, following the manufacturer's instructions (ebioscience Co., San Diego, CA, USA).

### 2.5. Pathological Evaluation of the Lung

The lung histological evaluation was performed as described previously (32). Briefly, the left lung was fixed in 10% buffered formalin (37%, Merck, Germany), and the lung specimens were embedded in paraffin, sectioned at 3-4 *μ*m thickness, and stained with hematoxylin-eosin (H&E) solution. Using a light microscope and based on the following scoring system, inflammation, hemorrhage, interstitial fibrosis, epithelial damage, and emphysema changes were evaluated in the lug specimens of different groups. Pathological insult scores were defined as follows: 0, no pathological changes; 1, patchy changes; 2, local changes; and 3, severe changes (in most parts of the lung) [26].

### 2.6. Statistical Analysis

Comparison among groups was made using one-way analysis of variance (ANOVA) with Tukey multiple comparison tests. Data were presented as mean ± SEM. InStat (GraphPad Software, Inc., La Jolla, USA) software was used for statistical analysis, and *P* < 0.05 was used as statistical significance criteria.

## 3. Results

### 3.1. Total and Differential WBC Counts in the Blood and the BALF

**Figure 1:**
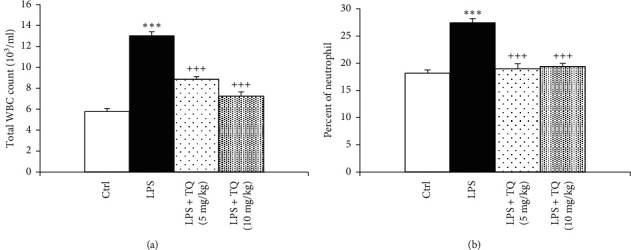
Total white blood cell count (a) and percent of neutrophils (b) in blood. Data are presented as means  ±  SEM (*n* = 6 in each group). ^*∗∗∗*^*P* < 0.001 vs. control group; ^+++^*P* < 0.001 vs. LPS group. For comparison between groups, one-way analysis of variance (ANOVA) with Tukey multiple comparison tests was used.

**Figure 2:**
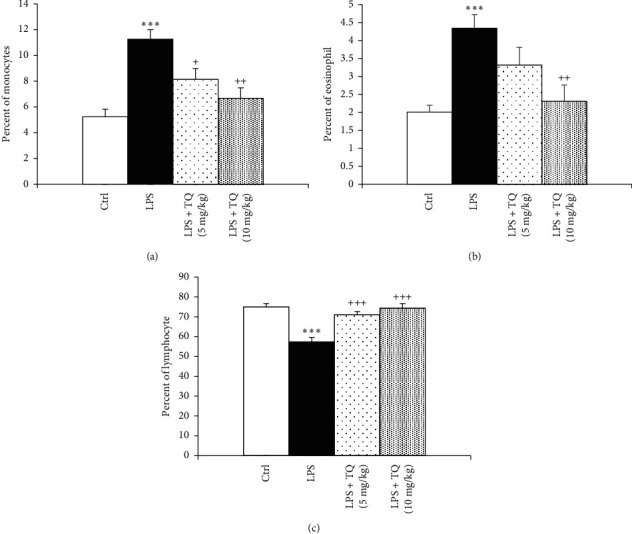
Percentages of monocyte (a), eosinophil (b), and lymphocytes (c) in blood. Data are presented as means ± SEM (*n* = 6 in each group). ^*∗∗∗*^*P* < 0.001 vs. control group; ^+^*P* < 0.05, ^++^*P* < 0.01, and ^+++^*P* < 0.001 vs. LPS group. For comparison between groups, one-way analysis of variance (ANOVA) with tukey multiple comparison tests was used.

**Figure 3:**
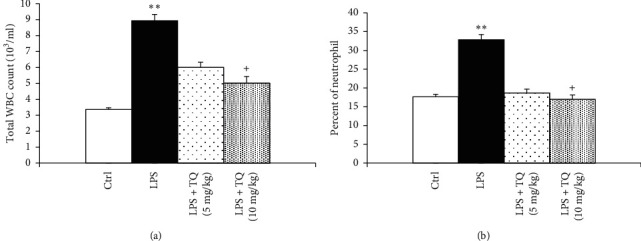
Total white blood cell count (a) and percent of neutrophils (b) in the BALF. Data are presented as means ± SEM (*n* = 6 in each group). ^*∗∗*^*P* < 0.01 vs. control group; ^+^*P* < 0.05 vs. LPS group. For comparison between groups, one-way analysis of variance (ANOVA) with tukey multiple comparison tests was used.

**Figure 4:**
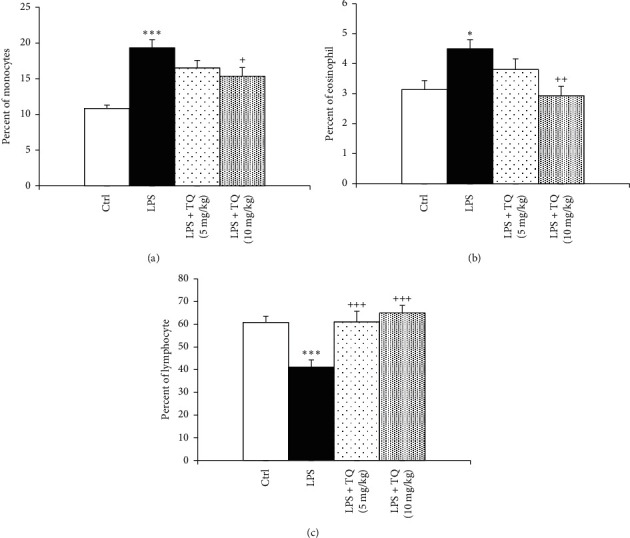
Percentages of monocyte (a), eosinophil (b), and lymphocytes (c) in the BALF. Data are presented as means ± SEM (*n* = 6 in each group). ^*∗*^*P* < 0.05, ^*∗∗∗*^*P* < 0.001 vs. control group, and ^+^*P* < 0.05, ^++^*P* < 0.01, and ^+++^*P* < 0.001 vs. LPS group. For comparison between groups, one-way analysis of variance (ANOVA) with tukey multiple comparison tests was used.

LPS administration resulted in significantly increased total WBC count and eosinophil, neutrophil, and monocyte percentages of in the both the blood and the BALF but a significant decrease in the lymphocyte percentage compared to the control group (*P* < 0.05 to *P* < 0.001). In the blood, total WBC count and percentages of neutrophil and monocyte were decreased, but lymphocyte percentage in the both blood and BALF was increased in treated groups with both doses of TQ. However, eosinophil percentage in the blood as well as total WBC count and percentages of eosinophil, neutrophils, and monocytes in the BALF were reduced and in rats treated with the higher dose of TQ compared to the LPS group (*P* < 0.05 to *P* < 0.001) (Figures 1–4).

### 3.2. Cytokine Levels in the BALF

**Figure 5:**
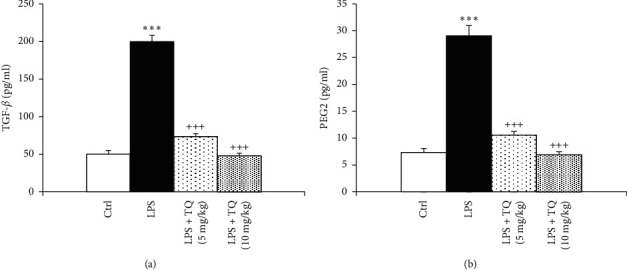
The concentrations of TGF-*β*1 (a) and PGE2 (b) in the BALF. Data are presented as means ± SEM (*n* = 6 in each group). ^*∗∗∗*^*P* < 0.001 vs. control group; ^+++^*P* < 0.001 vs. LPS group. For comparison between groups, one-way analysis of variance (ANOVA) with Tukey multiple comparison tests was used.

**Figure 6:**
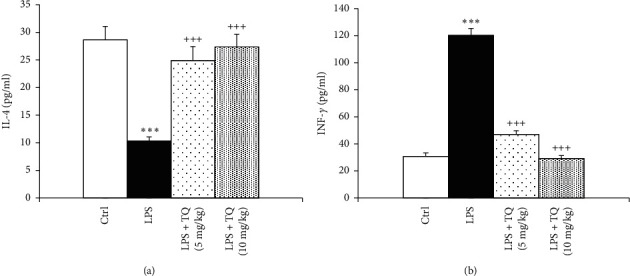
The concentrations of IL-4 (a) and INF-*γ* (b) in the tissue. Data are presented as means ± SEM (*n* = 6 in each group). ^*∗∗∗*^*P* < 0.001 vs. control group; ^+++^*P* < 0.001 vs. LPS group. For comparison between groups, one-way analysis of variance (ANOVA) with tukey multiple comparison tests was used.

Levels of PGE2, TGF-*β*1, and INF-*γ* were significantly enhanced, but IL-4 was significantly reduced in the BALF of the LPS-administered group as compared to the control rats (*P* < 0.001 for all cases). Treatment with both doses of TQ significantly reduced PGE2, TGF-*β*1, and INF-*γ* but increased IL-4 levels compared to the LPS group (*P* < 0.001 for all cases) (Figures 5 and 6).

### 3.3. Lung Pathology

**Figure 7:**
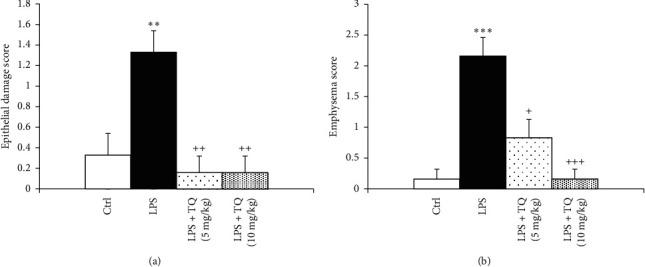
Epithelial damage (a) and emphysema (b) scores of lung tissue. Data are presented as means ± SEM (*n* = 6 in each group). ^*∗∗*^*P* < 0.01 vs. control group; ^++^*P* < 0.01 vs. LPS group. For comparison between groups, one-way analysis of variance (ANOVA) with tukey multiple comparison tests was used.

**Figure 8:**
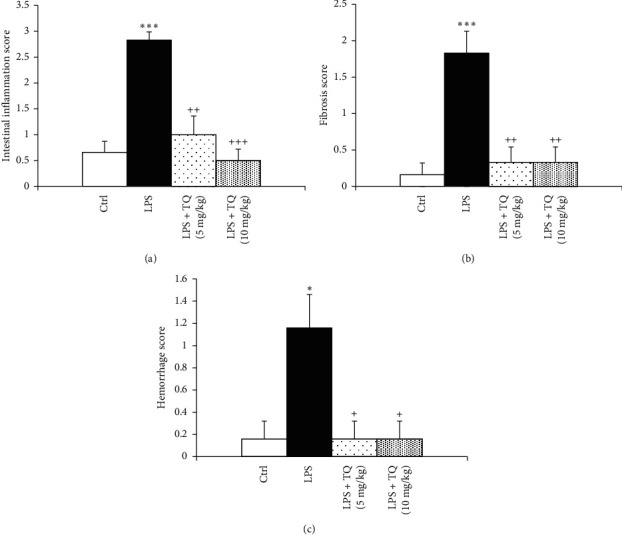
Intestinal inflammation (a), fibrosis (b), and hemorrhage (c) scores of lung tissue. Data are presented as means ± SEM (*n* = 6 in each group). ^*∗*^*P* < 0.05 and ^*∗∗∗*^*P* < 0.001 vs. control group; ^++^*P* < 0.01 and ^+++^*P* < 0.001 vs. LPS group. For comparison between groups, one-way analysis of variance (ANOVA) with Tukey multiple comparison tests was used.

**Figure 9:**
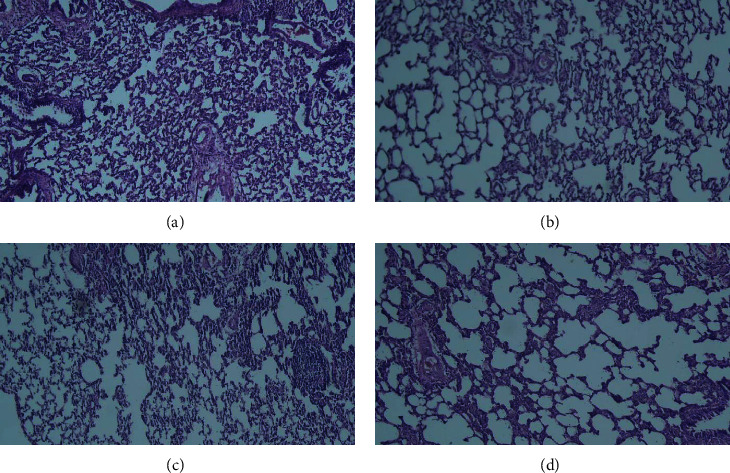
Representative photographs of pathological changes of lung in LPS-induced lung in the control group (a), LPS-exposed group (b), and LPS-exposed and -treated animals with low (c) and high (d) doses of TQ (hematoxylin and eosin staining, magnification 200x).

In the LPS group, pathological insults in the lung including fibrosis, interstitial inflammation, hemorrhage, epithelial damage, and emphysema were significantly increased (*P* < 0.05 to *P* < 0.001). Treatment with both TQ doses significantly ameliorated all pathological changes of the lung induced by LPS (*P* < 0.05 to *P* < 0.001) (Figures 7–9).

## 4. Discussion

The effect of TQ on lung injury induced by LPS in rats and the possible mechanisms underlying this effect were examined in this study. Previously, the anti-inflammatory effect of LPS was shown in the lung of animal models [27]. In this study, LPS injection for two weeks increased total and differential WBC counts in the BALF and the blood. Furthermore, LPS decreased the IL-4 level but increased IFN-*γ*, TGF-*β*1, and PGE2 levels in the BALF. Moreover, pathological insults of the lung such as lung inflammation, epithelial damage, emphysema, and fibrosis confirmed LPS-induced lung injury in the current study which was also shown in the previous studies [8, 10].

Based on the results of previous studies, chronic inflammatory processes in the lung were induced by long-term exposure to LPS [28]. Most studies showed that neutrophil infiltration into the lung tissue is an important inflammatory response factor of the lung to LPS administration [29]. In fact, bronchoalveolar neutrophilia was reported as the main cell response following LPS inhalation [30]. In the present study, increased total WBC count and percentages of eosinophils, neutrophils, and monocytes in the BALF and the blood showed systemic and lung inflammation induction. In the LPS-administered animals, decreased lymphocytes percentage was due to increased total WBC count. In fact, increased absolute lymphocytes count was demonstrated in previous studies [8, 10].

A previous study also showed systemic inflammation caused by the release of inflammatory cytokines following i.p. injection of LPS [8]. It was shown that LPS induced Th1 responses (IFN-*γ*) and inhibited Th2 responses (IL-4) through the toll-like receptor 4- (TLR4-) dependent pathway; also, LPS increased BALF levels of PGE2 and TGF-*β*1 [8]. TLR4 is the main receptor for LPS. The expression of TLR4 on mononuclear cells may be related to the exogenous LPS level and affects the balance of Th1/Th2 cells in the opposite way to that of LPS alone [27]. Therefore, the results of the current study in terms of systemic and lung inflammation due to chronic LPS administration are supported by the abovedescribed studies.

The emphysematous changes in lung architecture could occur due to chronic LPS exposure which can result from neutrophil infiltration, chronic inflammatory responses, airway wall thickening, mucus cell metaplasia, and irreversible alveolar enlargement [28, 31]. It is accepted that lung inflammation is the main characteristic of lung injury [8]. Pathological findings of the lung in the present study demonstrated that LPS induced lung injury, similar to the abovenoted studies.

The results of the present study showed a protective effect of TQ on LPS-induced lung inflammation dose dependently. Application of TQ decreased total and differential WBC counts in the blood and inhibited neutrophil, eosinophil, and monocyte infiltration into the airways when coadministered with LPS which indicated the improvement of systemic and lung inflammation. Administration of TQ increased Th2 response (IL-4), inhibited Th1 response (IFN-*γ*), and resulted in the improvement in Th1/Th2 immune homeostasis. Pretreatment with TQ caused considerable improvement of lung inflammation by decreased BALF levels of PGE2 and TGF-*β*1 as well as ameliorated pathological changes in the lung which all confirmed the protective effects of TQ on lung inflammation. However, the percentage of basophiles was very little to present their results in all studied groups both in the BALF and the blood. In addition, it was much more reliable if total and differential WBC counts in the blood and BALF were evaluated by flow cytometry, but their evaluation by the traditional method is still acceptable way and it was tried hard to avoid human errors in counting total and differential WBC.

The anti-inflammatory effect of TQ on lung inflammation in animal models of asthma was previously reported [12, 14–16, 32]. Evidence suggests that TQ decreases airway inflammation by stimulating IFN-*γ* secretion (Th1-mediated cytokine), inhibiting IL-4, IL-5, and IL-13 secretion (Th2-mediated cytokines), NF-*κ*B, IL1*β*, TNF*α*, and cyclooxygenase (COX)-1 expression, and PGE2 and PGD2 production, leukotriene (LT) B4 and LTC4 synthesis and eosinophil infiltration into the airways [15, 16, 32–34]. Furthermore, histopathological examinations, in previous reports, confirmed the antifibrotic effect of TQ [35, 36]. The ameliorative effect of TQ, mediated via cytokines rearrangement, was also indicated on LPS-induced pulmonary blood vascular damage in rats [37].

In this study, TQ not only reduced infiltration of WBCs, especially neutrophils, as one of the main component of the inflammatory response, into the lung but also decreased total and differential WBC counts in the blood and, thereby, reduced systemic inflammation.

The exact mechanisms of the anti-inflammatory activity of TQ in LPS-induced lung injury are not clear. TQ was shown to inhibit eicosanoid production and reduce proinflammatory lipid mediators in *vitro*. COX‐1 is a constitutively expressed enzyme with various physiological functions. TQ was shown to inhibit the synthesis of PGE2 by COX-1 and COX-2, in *vitro* [32]. This study suggests that the anti-inflammatory activity of TQ is probably mediated by a mechanism that targets COX-2-mediated PGE2 synthesis.

This study showed that TQ inhibited Th1 cell-mediated response and IFN-*γ* production as well as shifted the immune response toward a Th2-dominant pattern and IL-4 production. In this study, TQ-induced IL-4 production and Th2 cell-mediated response which probably play a key role in damping inflammation and LPS-induced lung injury.

Three isoforms of TGF-*β* as a master switch in the fibrotic process including TGF-*β*1, TGF-*β*2, and TGF-*β*3 existed in mammals. TGF-*β*1 is the isoform which is most closely related to the development of idiopathic pulmonary fibrosis [38]. It was indicated that TGF-*β* overexpression and TGF-*β*-smad3 signaling are implicated in pulmonary fibrosis and emphysema in experimental rodent models [39]. In the current study, TQ reduced TGF-*β*1 level and prevented the development of lung fibrosis and emphysema in LPS-induced lung injury. In fact, the effects of TQ in pulmonary vascular damage induced by LPS via cytokine downregulation in rats [37] and its effect on activated BV-2 microglial cells by LPS via reduction of NO2 and iNOS protein expression and improvement in various cytokines such G-CSF, MCP-5, MCP-1, and IL-6 protein in activated BV-2 cells [40] supporting the results of the current study were reported. The inhibitory effects of TQ on IL-4 production, OVA-specific IgE, TNF-*α* and IL-1b gene expression, edema, and eosinophil infiltration in the nasal mucosa were reported in a rat model of allergic rhinitis [41]. Antinociceptive and anti-inflammatory effects of TQ such as radical scavenging activity and interaction with proinflammatory enzymes and cytokines were indicated [42]. The effect of TQ on biosynthesis of various inflammatory mediators (5-LO, COX, PGD2, and LTs), reducing proinflammatory cytokines (interleukins (ILs) and TNF-*α*), reducing oxidative stress, and increasing chemokinesis, chemotaxis, phagocytic activity, antibody levels, and the hemagglutination, was also shown [43].

In this study, administration of TQ before LPS-induced inflammation showed apreventive effect on the development of lung inflammation. Therefore, it remains to be investigated whether TQ has a similar anti-inflammatory activity in the presence or after the induction of lung inflammation. Although a large number of studies have been published on anticancer and anti-inflammatory effect of TQ in the last 5 years, this study examined the effect of TQ in LPS-induced (i.p. administred) lung injury with different processes compared to previous studies for the first time.

Total and differential WBC in the blood as an indicator of systemic inflammation as well as total and differential WBC in BALF, BALF levels of various cytokine, and lung pathological changes as indicators of lung injury were measured in this study. However, in further studies, more precise mechanisms of LPS-induced lung injury and the protective effect of TQ including more inflammatory and oxidative stress markers should be evaluated.

## 5. Conclusions

The preventive effect of TQ on LPS-induced inflammation and lung injury was shown by reducing total and differential WBC airway inflammatory cells, lung hemorrhage, interstitial inflammation, epithelial damage and emphysema, fibrosis, and balancing Th1/Th2 immune response.

## Figures and Tables

**Table 1:** Studied groups, lipopolysaccharide (LPS) administration and thymoquinone (TQ) treatment.

Group	Saline or LPS administration	TQ treatment	Abbreviation
Control	Saline, 2 ml/kg/day, i.p. for two weeks	Saline, 30 min before LPS administration, i.p.	C
LPS administrated	LPS, 1 mg/kg/day, i.p. for two weeks	“	LPS
LPS + TQ 5 mg/kg	“	TQ, 30 min before LPS administration, i.p.	LPS-TQ 5
LPS + TQ 10 mg/kg	“	“	LPS-TQ 10

LPS and TQ were purchased from Sigma Chemical Co. LPS was dissolved in warm sterile saline supplemented with ethanol, and TQ was dissolved in cold sterile saline both freshly prior to injection. The doses of TQ [17, 18] and LPS [12–15] were chosen according previous studies.

## Data Availability

The data are available on request from the corresponding author Mohammad Hossein Boskabady (boskabadymh@mums.ac.ir).
